# Study on efficacy and safety of Huangqi Guizhi Wuwu decoction treatment for oxaliplatin induced peripheral neurotoxicity: A protocol for a randomized, controlled, double-blind, multicenter trial

**DOI:** 10.1097/MD.0000000000019923

**Published:** 2020-05-29

**Authors:** Xiao-Man Wei, Xiao-Feng Chen, Peng Shu, Zhi-Wei Jiang, Xiao-Yu Wu, Xi Zou, Kai Chen, Bo Shen, Wen-Wei Hu, Wei Lu, Wei-Xing Shen, Liu Li, Jun-Yi Wang, Feng-Jiao Zhao, Qing-Feng Yin, Hai-Bo Cheng, Yan-Hong Gu

**Affiliations:** a The First Clinical College of Nanjing University of Chinese Medicine; b Jiangsu Collaborative Innovation Center of Traditional Chinese Medicine Prevention and Treatment of Tumor; c Department of Oncology, The First Affiliated Hospital With Nanjing Medical University; d Department of Oncology, Pukou Branch Hospital of Jiangsu Province Hospital (Nanjing Pukou Central Hospital); e Department of Oncology, Affiliated Hospital of Nanjing University of Chinese Medicine; f Department of General Surgery, Affiliated Hospital of Nanjing University of Chinese Medicine; g Department of Surgical Oncology, Affiliated Hospital of Nanjing University of Chinese Medicine; h Department of Oncology, The First Affiliated Hospital of Soochow University, Suzhou; i Department of Oncology, Jiangsu Cancer Hospital; j Department of Oncology, The Third Affiliated Hospital of Soochow University, Changzhou; k Jiangsu Famous Medical Technology Co., Ltd, Nanjing University of Chinese Medicine, Nanjing, China.

**Keywords:** clinical trial, Huangqi Guizhi Wuwu decoction, oxaliplatin induced peripheral neurotoxicity, protocol, randomized

## Abstract

**Background:**

Oxaliplatin can cause severe peripheral neurotoxicity, which is an important reason for clinical oxaliplatin reduction and cessation of treatment. Oxaliplatin induced peripheral neurotoxicity (OIPN) can cause paresthesia and dysesthesia, even affect the quality life of patients. So far, there are no recognized and effective measures to prevent OIPN. Huangqi Guizhi Wuwu decoction is a classical prescription of ancient Chinese medicine recorded in “the synopsis of the Golden Chamber,” which can be used in the treatment of various neurotoxicity. However, there is a lack of large-scale and high-quality clinical studies on the prevention of OIPN by Huangqi Guizhi Wuwu decoction. The purpose of this study is to evaluate the efficacy and safety of Huangqi Guizhi Wuwu decoction on preventing OIPN.

**Methods/design:**

This study is a randomized, controlled, double-blind, and multicenter clinical trial. Three hundred sixty patients will be randomly assigned into Huangqi Guizhi Wuwu decoction group and Huangqi Guizhi Wuwu decoction mimetic agent group. Patients will receive chemotherapy with FOLFOX of 8 cycles of 3 weeks with Traditional Chinese Medicine (TCM) for 6 months and 1-year follow-up. The primary outcome measure is the differences in the incidence of chronic neurotoxicity of grade 2 and above during and after treatment. The secondary outcome measure is the improvement in other symptoms associated with chemotherapy. Four methods will be used to evaluate the efficacy of neurotoxicity, including oxaliplatin specific toxicity grading standard (Levi classification); CTCAE4.02 version; EORTC QLQ-CIPN20 scale, EORTC QLQ C30 scale, and EORTC QLQ-CR29 scale are used at the same time; Electromyography.

**Discussion:**

This study will provide objective evidences to evaluate the efficacy and safety of Huangqi Guizhi Wuwu Decoction on preventing OIPN.

**Trial registration:**

Clinical Trials.gov (Identifier: NCT04261920).

## 1 Introduction

Oxaliplatin is one of the most commonly used chemotherapeutic drugs in clinic. It is effective in ovarian, breast cancer, gastric cancer, pancreatic cancer, non-small cell lung cancer, melanoma, testicular, and lymphoma. More than 70% of patients with gastrointestinal tumors need oxaliplatin chemotherapy. Peripheral neurotoxicity is the main dose-limiting toxicity. About 90% of all patients treated with oxaliplatin have characteristic neurotoxicity.^[1]^ OIPN can be divided into acute neurotoxicity and chronic neurotoxicity (also as known as cumulative neurotoxicity). Transient peripheral neuropathy is the most common acute neurotoxicity in clinic, which is characterized by paresthesia at the end of the limb and hypersensitivity induced or aggravated by cold stimulation.^[2]^ Other acute neurotoxic symptoms include abnormal sensation in the throat, soreness around the mouth, mandibular spasm, and muscle spasm.^[3,4]^ The occurrence and severity of chronic peripheral neurotoxicity depend on the cumulative dose of oxaliplatin. Chronic neurotoxicity usually occurs in patients receiving the dose of oxaliplatin >540 mg/m^2^, characterized by sensory numbness and dullness in the distal limbs, and may affect daily activities, including writing, holding, and other fine movements.^[5]^ The MOSAIC study showed that when patients received the dose of oxaliplatin as 1000 mg/m^2^, 58% of patients developed grade 2 neurotoxicity (affecting daily life) and 18% developed grade 3 neurotoxicity (seriously affecting daily life). The patient recovers very slowly after developing grade 3 of cumulative neurotoxicity. In the MOSAIC study, the median time for patients to recover from grade 3 neurotoxicity to grade 0 was >70 weeks.^[6]^ Therefore, neurotoxicity is an important factor that limits the clinical application of oxaliplatin and affects the quality of life of tumor patients.

Many drugs are also used to try to prevent or reduce OIPN, including acetylcysteine, amifostine, calcium-magnesium mixtures, glutathione, ORG2766, gabapentin, vitamin E, as well as antidepressants and antiepileptic drugs. The 2014 ASCO guidelines^[7]^ reported the results of 48 clinical studies of chemotherapy-related neurotoxicity, only 1 study using duloxetine had positive results, and found that duloxetine reduced chemotherapy-related pain. However, duloxetine is a drug for the treatment of severe depression, with numerous and obvious side effects in cardiovascular, central nervous system, endocrine system, reproductive system, and gastrointestinal system. In 2019, Professor Yuanhong et al^[8]^ conducted a study on GM1 significantly reducing the peripheral neurotoxicity induced by paclitaxel in the treatment of breast cancer, which is the only multicenter double-blind phase III randomized controlled clinical trial that has proved to be effective in preventing neurotoxicity caused by chemotherapy. Therefore, looking for effective drugs to prevent OIPN is still an important problem to be solved.

Huangqi Guizhi Wuwu decoction, listed in Table 1, is recorded in the synopsis of the Golden Chamber, “both yin and yang are weak, the pulse on the upper of cunkou region is faint, the pulse on the middle of chi area is small and tight, the body is numb in external syndrome like wind impediment. It should be treated by Huangqi Guizhi Wuwu Decoction.” According to its clinical manifestation, it belongs to the category of “blood arthralgia” in TCM. According to the records of the Canon of Internal Medicine, its pathogenesis is weakness of Rong Wei, invasion of exogenous pathogenic factor, paralysis of flesh and blood collaterals. The clinical manifestation is the paralysis of the body skin. There are many convergences between the blood arthralgia and the occurrence and development of neurotoxicity caused by oxaliplatin in clinical symptoms. Therefore, Huangqi Guizhi Wuwu decoction is often used to prevent OIPN in clinic.

**Table 1:**
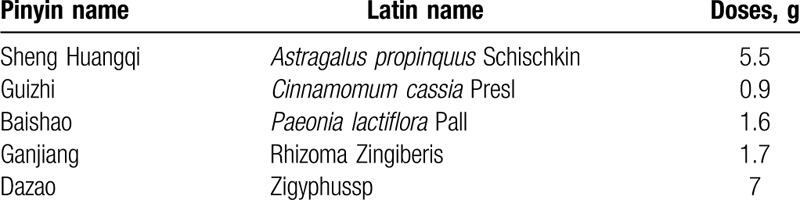
Standard formulation of Huangqi Guizhi Wuwu decoction.

## 2 Methods/design

### 2.1 Design

This study is designed as a randomized, controlled, double-blind, and multicenter trial to evaluate the efficacy and safety of Huangqi Guizhi Wuwu decoction on preventing OIPN. The trial was registered at ClinicalTrials.gov (NCT04261920), and protocol version is version 1.2/20191230. The clinical study will last 36 months and begin from January 2020 to January 2023.

**Figure 1:**
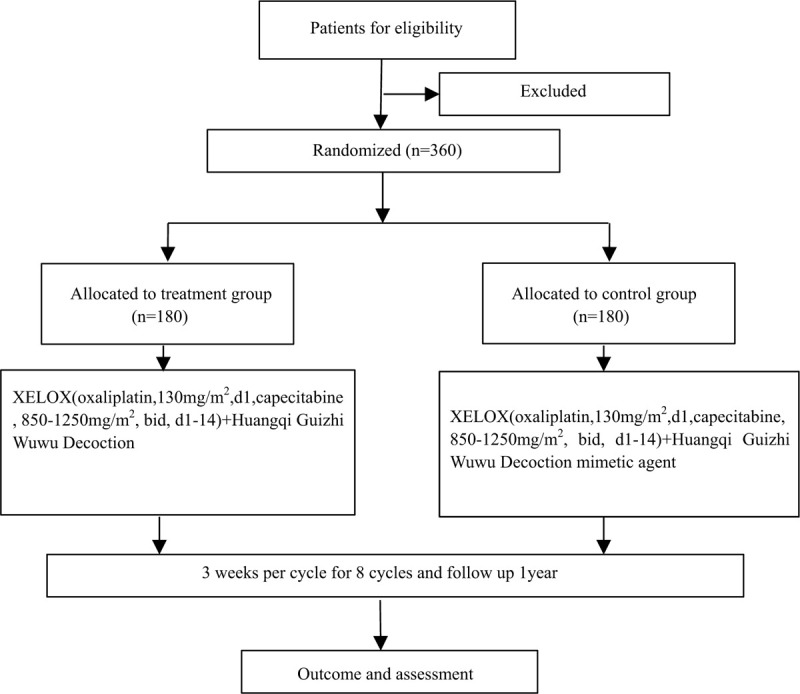
Study flow chart of enrollment, allocation, intervention, and assessment.

This study will be conducted in 13 clinical centers in China, including Affiliated Hospital of Nanjing University of Chinese Medicine, The First Affiliated Hospital With Nanjing Medical University, Jiangsu Cancer Hospital, Pla Bayi Hospital, The First affiliated Hospital of Soochow University, The Second affiliated Hospital of Soochow University, Suzhou Municipal Hospital, Nantong Cancer Hospital, The first people's Hospital of Changzhou, The second people's Hospital of Changzhou, will recruit 30 patients through posters, Zhangjiagang first people's Hospital, Jiangyin People's Hospital, Pukou branch hospital of Jiangsu Province Hospital. A total of 360 patients will be recruited in all clinical centers. Five phases will be consisted in the study: screening, allocation, intervention, end of intervention, and follow-up. Each patient will give their written informed consent form prior to enrollment. The study's flow chart is shown in Fig. 1.

### 2.2 Randomization and allocation

Block randomization and center-based stratification was carried out in this study. With the help of SAS statistical software, the “random number table of central coding” was generated for a given number of seeds, and the subjects were divided into Huangqi Guizhi Wuwu decoction group and Huangqi Guizhi Wuwu decoction mimetic agent group according to the random number (the ratio of the 2 groups was 1:1).

### 2.3 Sample size

In the mosaic study, the incidence of neurotoxicity above grade 2 was 42%. It is assumed that the incidence of neurotoxicity in the test group is 15% lower than that in the control group, 150 cases in each group are required under 80% control. Considering 20% off rate, it is expected 180 cases in each group. Therefore, we will enroll the total sample size of 360 patients for this trial.

### 2.4 Eligibility criteria

Inclusion criteria:

(I) Subject with colorectal cancer diagnosed by histopathological examination; subject after radical resection of colorectal cancer confirmed by histopathological examination, staged as high-risk II stage (according to CSCO guidelines for diagnosis and treatment of colorectal cancer) or III stage, IV stage (metastatic lesions have been radical resection);
(II) Subject who is suitable for receiving oxaliplatin plus capecitabine regimen as adjuvant chemotherapy for 6 months, the cumulative dose of oxaliplatin is expected to exceed 520 mg/m^2^ at least;
(III) Subject is between 18 and 75 years of age, men or women;
(IV) ECOG score ranges from 0 to 1
(V) Seven days before treatment, the functions of major organs (heart, liver, kidney, bone marrow) meet the following criteria:
  Standard of blood routine examination (without blood transfusion within 14 days)
  i. Hemoglobin (HB) ≥90 g/L;
  ii. Absolute neutrophil count (ANC) ≥1.5 × 109/L;
  iii. Platelet (PLT) ≥80 × 109/L.
  Biochemical examination should meet the following standards:
  i. Total bilirubin (TBIL) ≤1.5 times upper normal limit (ULN);
  ii. Alanine aminotransferase (ALT) and aspartate aminotransferase (AST) ≤2.5 times ULN;
  iii. Serum creatinine (Cr) ≤1.5 times ULN or creatinine clearance (CCr) ≥60 mL/min;
(VI) Expected survival time ≥12 months;
(VII) For subjects who have used other chemotherapeutic drugs in the past, they need to go through a clearance period of at least 4 weeks before entering this trial.
(VIII) The subjects who have signed the informed consent form.

Exclusion criteria:

(I) Subject had original nervous system diseases, including peripheral neuropathy and central neuropathy;
(II) Subject who are allergic to oxaliplatin or the ingredients of this traditional Chinese medicine;
(III) Clinical symptoms of subject with severe damp-heat syndrome of colorectal cancer include: dry mouth, bitter taste in the mouth, sticky sensation in mouth, yellow urine, dry stool, red tongue with yellow, thick and greasy fur;
(IV) Subject had neurological disease caused by electrolyte disorders or diabetes;
(V) Subject had symptoms of nerve compression caused by various causes;
(VI) At the same time, subject received other neuroprotective therapy, including nerve growth factor, vitamin B and calcium-magnesium mixture;
(VII) Subject was treated with oxaliplatin for chemotherapy before;
(VIII) Subject needed radiotherapy within half a year after operation;
(IX) Pregnant or lactation period women;
(X) Subject had cognitive impairment or psychosis;
(XI) Other subjects the investigator considers unsuitable for inclusion.

## 3 Interventions

### 3.1 Huangqi Guizhi Wuwu decoction granule group

Participants in the Huangqi Guizhi Wuwu decoction granule group will be treated with Huangqi Guizhi Wuwu decoction.

Participants will have been taking Huangqi Guizhi Wuwu decoction during adjuvant chemotherapy with XELOX regimen for 6 months (3 weeks per cycle for 4 cycles). Huangqi Guizhi Wuwu decoction will be taken twice a day, infused with warm water, 1 hour after lunch and dinner. One course of treatment will take 21 days. Participants will be contacted by telephone every one cycle and queried regarding adherence to study agents, illnesses, medication, and supplement use. The assessment that needs to be performed at visit is listed in Table 2.

**Table 2:**
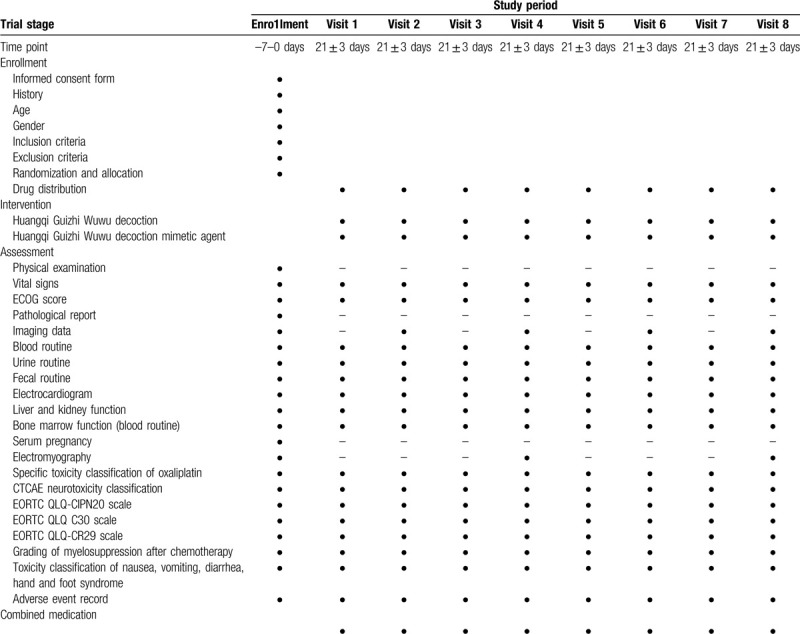
Study phases schedule for enrollment, intervention, and assessment.

### 3.2 Huangqi Guizhi Wuwu decoction mimetic agent group

Huangqi Guizhi Wuwu decoction mimetic agent group's treatment and measurements are in accordance with the Huangqi Guizhi Wuwu decoction group.

### 3.3 Treatment cycles

Six months treatment period will be planned in 8 cycles of 3 weeks. Between each cycle we will return visit participants to evaluate the efficacy of neurotoxicity. Chronic neurotoxicity can only be evaluated after chemotherapy, once every 2 months and followed up for 1 year.

### 3.4 Intervention drugs and blinding

Intervention drugs are Huangqi Guizhi Wuwu decoction and Huangqi Guizhi Wuwu decoction mimetic agent, which are provided by Tianjiang Pharmaceutical Group Co. Ltd., Wuxi, China. The size, shape, color, taste, package, and Lot number between Huangqi Guizhi Wuwu decoction and the mimetic agent is same.

The work of setting up blindness is completed by statisticians of Jiangsu Famous Medical Technology Co., Ltd. and is divided into 2 levels. The first level of blindness is the blindness of experimental drugs, which are unified packaging. The secondary blindness is the number of the packaging box of experimental drugs.

Only when it is really necessary to break the code in case of emergency, the researcher shall consult the person in charge of the center. With the signature of the person in charge of the center, the emergency blind letter can be opened and recorded, and the clinical research unit shall be notified within 24 hours after the blindness is broken.

### 3.5 Drug combination

During this study, participants with grade 2 or more neurotoxicity should be allowed to receive necessary drug treatment and their use must be recorded in detail in the CRF. However, following medication should be prohibited before the termination of the trial: antiepileptic drugs, such as acarbazepine, gabapentin; calcium-magnesium mixture, such as calcium gluconate, magnesium sulfate; vitamins, such as vitamin B, vitamin E; nerve growth factor; amifostine; other traditional Chinese medicine.

### 3.6 Outcome evaluation

#### 3.6.1 Primary outcome

The primary outcome measure of this study is the differences in the incidence of chronic neurotoxicity of grade 2 and above during and after treatment.

#### 3.6.2 Secondary outcome

The secondary outcome measure is difference in the incidence of acute neurotoxicity; time of occurrence of chronic toxicity to grade 2 and 3; incidence and severity of main symptoms; recovery time of grade 2 and 3 neurotoxicity; cumulative dose of oxaliplatin and the proportion of patients who stop using oxaliplatin because of neurotoxicity; incidence of myelosuppression and incidence of nausea, vomiting, diarrhea, and hand-foot syndrome.

### 3.7 Safety evaluation

Up to date, there are no reports of adverse reactions in clinical application of Huangqi Guizhi Wuwu decoction. However, participants may have toxic reactions of hematopoiesis, digestive system, and nervous system when using oxaliplatin, and toxic reactions such as digestive system and skin may occur when using capecitabine. To monitor the adverse reactions when using chemotherapy, some check must be taken including routine examination, urine routine examination, liver function test, renal function test, bone marrow function test (blood routine), serum pregnancy test (women only), electromyography.

All adverse events, including toxicity and side effects such as toxic reactions of hematopoietic system, gastrointestinal system, and nervous system, should be recorded in the CRF in detail. When an adverse event occurs, the investigators should decide whether to stop the trial according to the severity, and the corresponding treatment should be given in time according to the patient's condition. In case of serious adverse events, immediate measures should be taken to protect the safety of the patients. Adverse events should be reported to each hospital.

### 3.8 Data management and monitoring

The data management of this trial is based on electronic data management system, and the data administrator constructs eCRF according to the trial scheme and case report form. The data entry operator can timely and accurately input the data in the case report form into eCRF. eCRF is not the original record. Its’ content is derived from the “case report form.” When all the subjects complete the study, all the case report forms are entered into the system, and after they are reviewed by the inspectors and checked by the data administrator, the data are locked by the data administrator. After all the data are locked, the data administrator will import it into the designated database and submit it to the statisticians for statistical analysis.

### 3.9 Statistical analysis

After the test trial is determined, the statistical professionals shall be responsible for drawing up the statistical analysis plan in consultation with the investigators. The database will be analyzed by SAS 9.4 and will cover actual number of subjects selected, shedding and exclusion of cases, demographics and other baseline characteristics, compliance, efficacy analysis, and safety analysis.

The qualitative data are analyzed by chi-square test, Fisher exact test, Wilcoxon rank sum test, and CMH chi-square test. The quantitative data accord with the normal distribution by *t* test, and do not accord with the normal distribution by Wilcoxon rank sum test and Wilcoxon signed rank sum test. The two-sided test is used in the hypothesis test, and the test statistics and their corresponding *P* values are given, with *P* ≤ .05 as statistically significant and *P* ≤ .01 as highly statistically significant.

### 3.10 Discussion

Oxaliplatin, a third generation platinum compound, is a key drug used for the treatment of advanced colorectal cancer.^[9]^ However, oxaliplatin causes severe peripheral neuropathy and is clinically compelling because of it. Peripheral neuropathy is a well-recognized dose-limiting toxicity of oxaliplatin^[10]^ and can be detrimental to the quality of life of cancer survivors.^[11]^ OIPN can result in dosage reductions or even cessation of chemotherapy. So far, several types of agents show the possible treatment for OIPN including calcium and magnesium infusions, glutathione, venlafaxine, and carbamazepine.^[12]^ However, there is no sufficient available data in double-blinded, placebo-controlled trials show that these agents have neuroprotective capacity against OIPN.^[13]^ More seriously, most of them could cause unexpected side-effects.

Huangqi Guizhi Wuwu decoction^[14]^ is mainly used for the treatment of blood arthralgia, enriching qi and warming meridian, and promoting arthralgia for blood circulation. The prescription is composed of Huangqi, Shaoyao, Guizhi, Shengjiang, and Dazao. In the prescription, Huangqi is monarch drug, which could greatly replenish Primordial Qi, assist energy to dispel evil, and solid the muscle surface. Guizhi warm meridian to activate yang, but also can disperse external evil. Compatible with Huangqi, they could supplement qi to warm yang, and nourish blood to restore normal menster. With the help of Huangqi, Guizhi invigorates qi to invigorate Wei yang; with the help of Guizhi, Huangqi solidifies the surface without leaving evil. Shaoyao can nourish blood and protect ying to dredge arthralgia. Shaoyao and Guizhi are both as minister drugs. Combined with each other, they can reconcile Ying Wei, dispel the evil wind on the surface. Shengjiang, as adjuvant drug, spreads wind evil and warm blood circulation to strength the effect of Guizhi because the disease lies on the surface of the skin. Dazao blends all kinds of medicine, matching with Shengjiang to help Guizhi and Shaoyao in order to regulate ying and wei. These traditional Chinese medicines, matching with each other to get rid of wind evil, regulate qi and blood, cure the arthralgia. The compatible characteristics of this prescription are invigorating qi and nourishing blood and warming meridians to disperse evil, so that the surface is fixed without leaving evil, and evil is dispelled without harming the body resistance. Some studies^[15,16]^ showed that Huangqi Guizhi Wuwu decoction could obviously improve blood circulation and promote the recovery of peripheral nerve.

Many scholars in China have conducted studies on the prevention of OIPN by Huangqi Guizhi Wuwu decoction and obtained positive results, but these studies are simple clinical observations with small samples, and there are no strict randomized controlled clinical trials with large samples. For example, Fei and Yuehua^[17]^ observed the clinical efficacy of modified Huangqi Guizhi Wuwu decoction in the prevention and treatment of acute peripheral neurotoxicity induced by oxaliplatin. Eighty patients were randomly divided into 2 groups. Forty patients in the experimental group were treated with modified Huangqi Guizhi Wuwu decoction at the same time. Forty cases in the control group were treated with chemotherapy alone. FOLFOX regimen was used in all chemotherapy regimens. Acute peripheral neurotoxicity was assessed with reference to Levi and Zhao Guoguang peripheral neurotoxicity score criteria of oxaliplatin. Results among the 40 cases in the experimental group, the incidence of acute peripheral neurotoxicity after oxaliplatin chemotherapy was about 40.0%. In 40 cases in the control group, the incidence of acute peripheral neurotoxicity was about 87.5%. There was significant difference between the 2 groups (*P* < .02), indicating that modified Huangqi Guizhi Wuwu decoction can effectively prevent acute peripheral neurotoxicity induced by oxaliplatin. Professor Cao Peng team^[18]^ extracted standardized decoction AC591 from Huangqi Guizhi Wuwu decoction and establish a rat model of peripheral neurotoxicity induced by oxaliplatin. The results showed that AC591 could reduce cold hyperalgesia, mechanical abnormal pain, and morphological damage of dorsal root ganglion induced by oxaliplatin. Conclusion showed that AC591 could prevent OIPN and didn’t decrease antitumor activity of oxaliplatin.

The aim of this study is to prove that whether Huangqi Guizhi Wuwu decoction can prevent and treat OIPN. So we have designed the protocol for a randomized, controlled, double-blind, multicenter trial that will provide evidence for the effectiveness and safety of Huangqi Guizhi Wuwu decoction treatment for OIPN.

### 3.11 Trial status

The trial was registered at ClinicalTrials.gov (NCT04261920), and protocol version is version 1.2/20191230. The clinical study will last 36 months and begin from January, 2020 to January 2023. The first patient was recruited at Jiangsu Province Hospital and 360 patients will be included in this clinical study.

## Author contributions

**Administrative support:** Yan-Hong Gu, Hai-Bo Cheng.

**Conception and design:** Yan-Hong Gu, Hai-Bo Cheng, Xiao-Feng Chen, Xiao-Man Wei.

**Collection and assembly of data:** Xiao-Man Wei, Wei-Xing Shen, Liu Li, Jun-Yi Wang, Feng-Jiao Zhao.

**Data analysis and interpretation:** Qing-Feng Yin.

**Final review approval of manuscript:** Xiao-Man Wei, Xiao-Feng Chen, Peng Shu, Kai Chen, Doctorf, Bo Shen, Wen-Wei Hu, Wei Lu, Wei-Xing Shen, Liu Li, Jun-Yi Wang, Feng-Jiao Zhao, Qing-Feng Yin, Hai-Bo Cheng Yan-Hong Gu.

**Manuscript writing:** Xiao-Man Wei, Xiao-Feng Chen, Peng Shu, Kai Chen, Doctorf, Bo Shen, Wen-Wei Hu, Wei Lu, Wei-Xing Shen, Liu Li, Jun-Yi Wang, Feng-Jiao Zhao, Qing-Feng Yin, Hai-Bo Cheng Yan-Hong Gu.

**Provision of study materials study materials or patients:** Peng Shu, Kai Chen, Bo Shen, Wen-Wei Hu.
